# Under-Vine Vegetation Mitigates the Impacts of Excessive Precipitation in Vineyards

**DOI:** 10.3389/fpls.2021.713135

**Published:** 2021-07-26

**Authors:** Justine Vanden Heuvel, Michela Centinari

**Affiliations:** ^1^School of Integrative Plant Science, Cornell University, Ithaca, NY, United States; ^2^Department of Plant Science, The Pennsylvania State University, University Park, PA, United States

**Keywords:** climate change, competition, cover crop, soil health, vigor, *Vitis*

## Abstract

Excessive precipitation events have greatly increased in several grape growing regions due to human-caused climate change. These heavy downpours result in a myriad of problems in the vineyard including soil aggregate breakdown, soil runoff, nutrient leaching, excessive vine vegetative growth, and diseased fruit. The negative impacts of excessive precipitation events on vineyards are exacerbated by the maintenance of bare soil under the vines. Exposure of bare soil results in soil erosion and runoff which pollutes nearby watersheds; raindrops weaken and break apart soil aggregates, leading to increased soil erosivity and contributing to the formation of surface crusts. In addition to excessive precipitation events, some grape growing regions can be characterized by fertile soils. The availability of ample water and nutrients can lead to highly vigorous vines with shoot growth continuing through harvest. Long shoots and large leaves result in shaded fruit, a humid vine microclimate, and excessive cluster rot. In this review, we examined how either natural (i.e., resident) or seeded under-vine vegetation (UVV) can help mitigate many of the problems associated with excessive precipitation. Through providing vegetative coverage to reduce the force of raindrops, increasing soil organic matter and enhancing soil microbial diversity, UVV can reduce the soil degradation and off-site impacts caused by excessive precipitation events. Through competition for soil resources, UVV can reduce excessive vegetative growth of vines and decrease cluster rot incidence and severity, although grapevine response to UVV can be highly variable. We discussed recent advances in understanding below and aboveground vine response and acclimation to UVV and presented current evidence of factors influencing the impact of UVV on vine growth and productivity to assist practitioners in making informed decisions and maximize the ecosystem services provided by UVV.

## Introduction

There has been increased interest in understanding grapevine response and acclimation to changes in water availability induced by climate changes in order to adapt management strategies. Although increased drought is a major agricultural challenge and considerable emphasis has been placed to ameliorate the impact of lower water availability on wine grape production worldwide, some grape growing regions are facing challenges relating to more erratic rainfall patterns and excess water availability. In general, heavy precipitation events (measured as observed change in total annual precipitation falling in the heaviest 1% of events) are becoming more intense and more frequent across most of the United States (U.S. Global Change Research Program, 2014). This increasing trend is particularly strong in the northeastern United States, where excessive precipitation events increased by 71% between 1958 and 2012. In this region, rainfall events greater than 150 mm over 24 h increased in frequency from six events between 1979 and 1996 to 25 events from 1997 to 2014, a 317% increase (Howarth et al., 2019). In central Europe, heavy precipitation, defined as the 95th percentile of daily precipitation, increased from 1 to 3 mm/day per decade in the last 100 years, while during the last 60 years extreme winter precipitation intensified by 6–8% per decade in western Europe (Zolina, 2012). Both observations and climate model projections indicate strong increases in extreme precipitation in northern Europe as well. Climate model projections also show increases in extreme precipitation and flood discharge in the 21st century throughout Europe (reviewed by Madsen et al., 2014). Hosseinzadehtalaei et al. (2020) suggest that the frequency of extreme precipitation events in Europe will be tripled under the representative concentration pathway (RCP8.5) high emissions global scenario.

These heavy precipitation events can lead to a multitude of detrimental impacts on plants and soil in vineyards, particularly those with a lack of soil cover, as well as their neighboring ecosystems. Exposure of bare soil results in greater impact from raindrops, which weaken and break soil aggregates apart, increasing the erosivity of soils and contributing to the formation of crusting on the soil surface (Epstein and Grant, 1973). In vineyards in central Spain, runoff from bare soil was more than three times higher than from soil with vegetation cover, while nitrates lost in the runoff were almost six times greater from bare soil than from covered soil (García-Díaz et al., 2017). Even in regions where vegetation cover between rows is a common practice, nitrogen (N) and dissolved organic carbon can leach at a greater rate from under-vine bare soil compared to vegetation-covered soil (Karl et al., 2016a). Runoff and leaching cause decreases in soil fertility and eutrophication in downstream bodies of water.

Grapevines themselves can also be detrimentally impacted by increased and/or excessive precipitation. An increase in plant available water – and hence nutrient availability – can lead to greater vegetative growth (Giese et al., 2015) and berry size (Karl et al., 2016b) as well as extended growth of shoot tips (Centinari et al., 2016). Increased vegetative growth can cause cluster rots through decreased air flow in the canopy, while increased cluster compactness (due to increased berry size) also contributes to fungal pressure on the cluster (Valdes-Gomez et al., 2008; Guilpart et al., 2017). Therefore, highly vegetative vines might require more extensive and costly management practices to remediate these potential issues.

In grape growing regions with high precipitation, where inter-row vegetation is already maintained, researchers have been experimenting with under-vine vegetation (UVV) to alleviate some of the detrimental effects to soil and plants caused by ample precipitation and provide further benefits to the vineyard ecosystem. Both annual and perennial cover crop species have been intentionally planted in the area beneath the vines, but adoption of natural vegetation (i.e., managed weed growth) has also been explored. In addition to the eastern United States, UVV has been trialed in a range of climates including wine regions in France (Delpuech and Metay, 2018), Spain (Abad et al., 2020), Australia (Penfold and Howie, 2019), and New Zealand (Merfield, 2019), where excessive precipitation might not be a concern.

This review details how UVV can help ameliorate climate challenges related to increased heavy precipitation; we focus on key soil and plant traits that could be impacted by implementing UVV, describe the current understanding of the complex UVV-vine interactions, and identify knowledge gaps in the published literature. Our discussions are intended to provide a framework that can guide future research and increase UVV adoption. Due to the common use of inter-row cover crops in many wine regions with high precipitation, we focus on the additional impact UVV provides in vineyards where inter-row vegetation is already maintained.

## Soil Health and Ecosystem Services

Protecting soil from degradation is important for the long-term sustainability of a vineyard. Introducing cover crops into what was traditionally considered a perennial monoculture system can help achieve this goal (Garcia et al., 2018). Cover cropping between rows has been extensively studied in vineyards around the world, but information on complete vineyard floor cover crops (between row plus under-row) is limited. Below we summarize how introducing UVV can positively influence important parameters of soil health. These results, however, should be maintained in the context of short-term effects, within 2 or 3 years from UVV establishment as scientific investigations are often limited to a few years of field data collection.

### Soil Organic Matter and Soil Carbon

**Figure 1:**
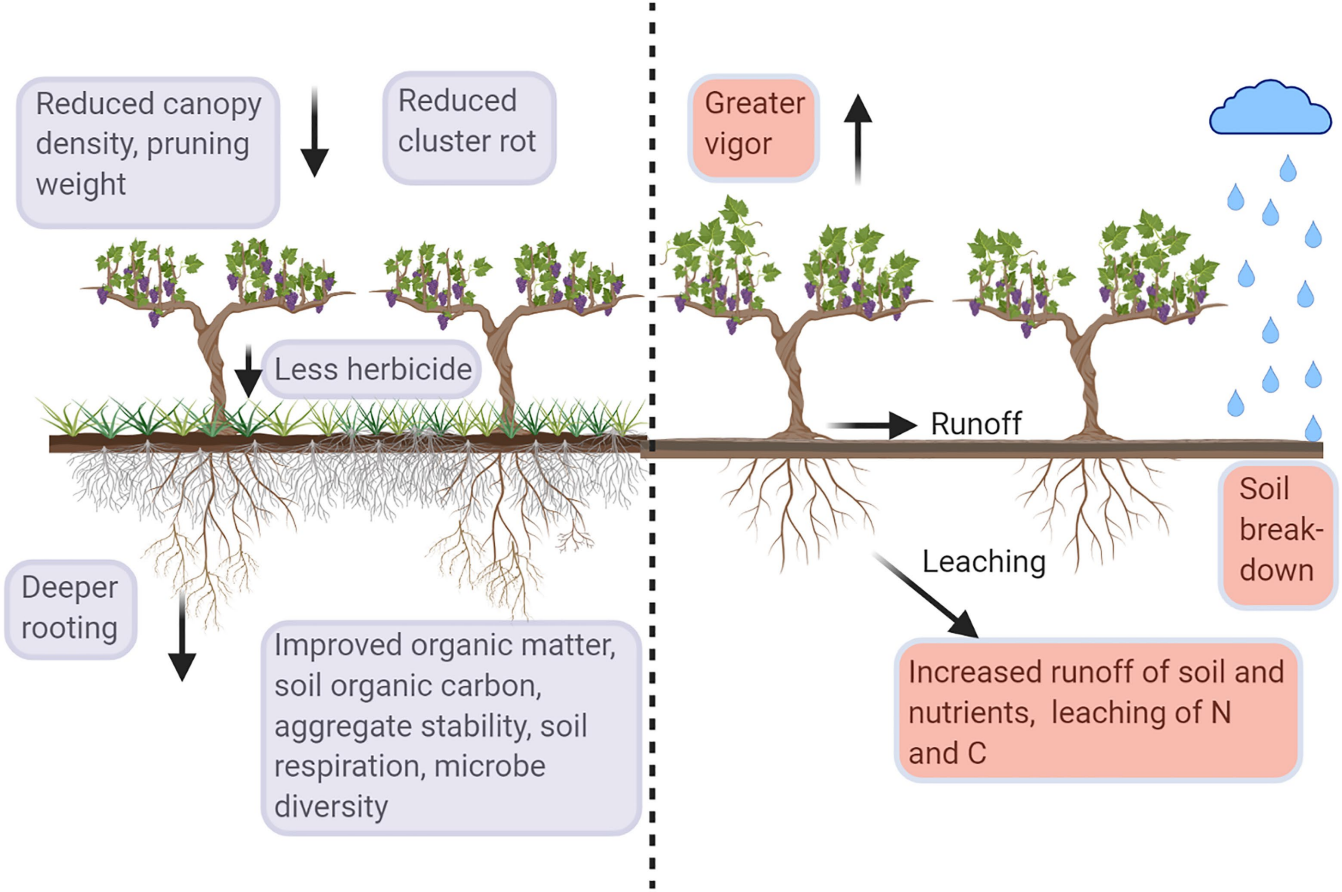
Diagrammatic representation of the impact of soil management, under-vine vegetation (UVV) vs. bare soil, on grapevines and soil. Violet boxes represent ecosystem services in vineyards with high-vigor potential; red boxes represent ecosystem disservices. Figure created with BioRender.com.

Soil organic matter (SOM), soil organic carbon (SOC, a component of SOM), and total carbon (C) can markedly increase or decrease as a function of soil management, although the response to management can take many years to become detectable. Repeated herbicide applications and cultivation result in bare soil, negatively impacting SOM and SOC (Figure 1). Plants can contribute biomass as well as rhizodeposits, directly increasing soil C. Indirectly, plants play a role in modifying soil C pools through microbial stimulation and reductions in soil erosion. Contributions of UVV to SOM and soil C are likely dependent on whether the vegetation is incorporated into the soil (e.g., annual species) or whether it remains in place over multiple years (e.g., perennial species). Working with annual species as UVV for 3 years, Karl et al. (2016a) reported an increase in SOM of only 0.6% compared to under-vine plots managed with herbicide. In a cold climate vineyard in Iowa, United States, changes in SOM were only apparent in shallow soil after 6 years of UVV treatments (DeVetter et al., 2015). Changes in SOM in vineyards with inter-row cover crops are also reported to be slow (Garcia et al., 2018).

An additional benefit of using cover crops in vineyards is the possibility of sequestering atmospheric C and N by increasing their concentration across the soil profile. A recent study analyzed changes in soil C and N across the under-vine soil profile (0–100 cm) 3 years after UVV (red fescue, *Festuca rubra*) was planted in a young vineyard (Fleishman et al., 2021). Planting an under-vine grass with a dense root system rather than maintaining bare soil significantly increased soil C by 56 and 44% at 1–20 cm and 21–40 cm soil depth, respectively. Total N in the UVV plots was 37 and 19% at 1–20 and 21–40 cm soil depth, respectively, higher than in the bare soil plots only after 3 years of vine-UVV coexistence. The increases in total C and N in the shallow soil layers were explained by UVV root biomass which colonized most of the shallow soil. It is unknown if over a longer time UVV will contribute to increases in deep soil C (ex. below 40 cm) in vineyards.

### Soil Physical Characteristics

Exposure of bare soil results in greater impact from raindrops, which weakens and breaks soil aggregates apart, increasing the potential of erosion and contributing to the formation of surface crusts (Epstein and Grant, 1973; Figure 1). Karl et al. (2016a) reported that using white clover (*Trifolium repens*) as an UVV for 4 years resulted in 36% greater aggregate stability than under-vine soils maintained with the herbicide glyphosate and 23% greater than those maintained with cultivation. Soils from the under-vine white clover plots maintained almost 75% of aggregate mass after a simulated rain event (Karl et al., 2016a). In a nearby vineyard, Chou and Vanden Heuvel (2019) reported increases of 82% in soil aggregate stability between glyphosate-maintained soil and natural vegetation (i.e., managed weed growth) after 3 years of UVV.

Based on the positive impact inter-row cover crops have had on improving water infiltration in vineyards (Celette et al., 2009), it is possible that UVV holds potential for improving soil infiltration through maintaining favorable soil structure and porosity, thereby reducing the opportunity for runoff along the soil surface following excessive precipitation. DeVetter et al. (2015) reported a massive but statistically insignificant increase in infiltration in a UVV treatment (creeping red fescue) compared to herbicide (0.6 min and 14.7 min for 444 ml of water to infiltrate the soil covered by UVV and maintained with herbicide, respectively) on a fine loam soil (DeVetter et al., 2015). However, Karl et al. (2016b) reported no differences in saturated soil infiltration rate among under-vine treatments of vegetation and bare soil over 4 years on a silt loam soil.

### Soil Microbial Activity

Microbial activity responds quickly to changes in soil management practices, often indicating changes in the flux of labile C before differences in SOM are apparent. Soil respiration is often used as a proxy for microbial activity of a soil, particularly when quantified in the absence of roots. Soil from UVV treatments had the greatest soil respiration rates in a number of studies (Figure 1). In the Finger Lakes region of New York, United States, soil respiration in the UVV natural vegetation treatment was 43% greater than in under-vine plots maintained with glyphosate and 45% greater than in plots maintained with cultivation (Karl et al., 2016a). In a nearby vineyard, soil respiration was 49% greater in under-vine plots with natural vegetation compared to those maintained with glyphosate, while plots with planted UVV, such as tall fescue (*Festuca arundinacea*), chicory (*Cichorium intybus*), tillage radish (*Raphanus sativus*), and alfalfa (*Medicago sativa*), had soil respiration rates up to 75% greater than plots maintained with glyphosate (Chou and Vanden Heuvel, 2019). The trend of greater soil respiration with UVV compared to herbicide or cultivation indicates that lack of vegetation decreases the input of biodegradable substrates to the soil, diminishing microbial activity and potentially lowering the rate of N mineralization into plant available forms (Rustad et al., 2001).

A more diverse community of soil microbes tends to be associated with decreased incidence of plant diseases as well as improved plant productivity (Vukicevich et al., 2016). The impact of floor management practices on vineyard microbiome has been overlooked until recently but is of significant interest as soil may be considered the vineyard microbial pool (Zarraonaindia et al., 2015). As UVV expands the diversity of plants in the vineyard, microbes associated with those herbaceous plants can broaden overall soil microbial diversity. In California, vineyard floor management impacted the composition of soil bacteria, but a potential association between soil, rhizosphere, and fruit microbiome was not investigated (Burns et al., 2016). In the cool, wet climate of upstate New York, Chou et al. (2018) studied the impact of three under-vine practices (herbicide application, soil cultivation, and natural vegetation as UVV). The authors reported that soil bacterial and fungal composition in the UVV treatment differed from the plots maintained with glyphosate (Chou et al., 2018). Although several studies have proved that cover crops planted either in the inter-row or under-vine area can affect the soil microbiome pool, we are still far from understanding the subsequent impact on vine functioning and productivity.

### Additional Ecosystem Services/Disservices

Other off-site impacts of concern in regions where vineyards are predominantly located on slopes – particularly in close proximity to bodies of water – are runoff and leaching of nutrients and agrochemicals (Figure 1). Lack of soil cover can increase the severity of soil runoff (Battany and Grismer, 2000). While the additional contribution of UVV to inter-row cover crops on soil runoff has not been directly quantified, UVV presumably provides a physical barrier that further reduces runoff when rows are planted perpendicular to hillsides. Greater dissolved SOC leaching from under-vine soils in comparison with those with UVV was reported by Karl et al. (2016a), indicating C loss from the agroecosystem. Total N leaching was great in the glyphosate-maintained plots as well as the legume white clover plots. Other vineyard groundcover studies found greater N leaching in herbicide-treated inter-rows, although this result is not simply a function of bare soil as cultivated plots had lower leaching of N (Steenwerth and Belina, 2010). A greater presence of soil C, microbial biomass, and plant residues has been linked with reduced N leaching in vineyard systems (Steenwerth and Belina, 2008). Greater leaching of nitrate can lead to increased emissions of the greenhouse gas nitrous oxide from soils (Steenwerth and Belina, 2010).

In a recent review, Garcia et al. (2018) summarized the important role cover crops play in weed control, pest and disease status, water availability, field trafficability, soil biodiversity, and C sequestration. These impacts are defined as ecosystem services, which are the conditions and processes through which ecosystems sustain human life. In Mediterranean-climate regions, competition for soil resources, chiefly water and nutrients, between the cover crops and grapevine is typically viewed as an ecosystem disservice because it can negatively suppress vegetative growth, reduce yield potential, and fruit composition (Garcia et al., 2018). However, the balance between service and disservice is dynamic and the ability of cover crops to provide ecosystem services or disservices varies depending on climate and soil conditions, the species of cover crop, as well as the coverage of the soil among other factors. For example, competition for soil resources from complete vineyard floor cover might provide a beneficial regulation of vegetative growth in a region and/or season with high precipitation and be considered an ecosystem service rather than a disservice. In other instances, less competitive cover crops (for example, annual herbaceous species or leguminous species) can be used as UVV to provide ecosystem services while limiting potential effects on vine growth and production (Jordan et al., 2016; Abad et al., 2020).

Another example of an important ecosystem service provided by cover crops is the biological control of pests. Beneficial insect presence in an ecosystem is usually positively correlated with vegetation abundance and diversity (Letourneau et al., 2011). Between-row cover crops can enhance populations of natural enemies of pests, reducing spider mite and some leafhopper populations on grapes (Costello and Daane, 2003; English-Loeb et al., 2003). The impact of UVV on vineyard pests has not been directly investigated, although Wolf and Giese (2020) warn of potential vole and cutworm damage in UVV plots. Research on UVV impacts on diseases has been preliminary; a reduction in gray mold (*Botrytis cinerea*) was recorded when grapes were harvested from plots managed with UVV rather than bare soil (Coniberti et al., 2018b).

## Grapevine-UVV Interaction

### Aboveground Growth and Yield Responses

In addition to ecological benefits, UVV can be planted in vineyards to limit root uptake capacity and decrease vine growth. However, vegetative growth and yield reductions are not easily predicted. Grapevine-UVV interaction can produce a wide range of aboveground effects from no influence (Jordan et al., 2016) to significant reductions in vegetative growth (Hatch et al., 2011; Giese et al., 2014; Karl et al., 2016b; Coniberti et al., 2018a; Fleishman et al., 2019). While lower vegetative growth is often considered an ecosystem service in regions with ample precipitation (Giese et al., 2014; DeVetter et al., 2015; Hickey et al., 2016), it might potentially become a disservice if growth reductions are considered excessive (Karl et al., 2016b). Many factors affect responses of grapevines to UVV competition, including soil resource availability and demand from both plant species. Even within the same site, vegetative growth and yield reductions can fluctuate with annual shifts in resource availability and vine acclimation strategies (Giese et al., 2014; Hickey et al., 2016). Grapevine demand for water and nutrients is influenced by environmental conditions but also endogenous factors, such as vine age and rootstock (or root system genotype), which in turn affect root system volume and uptake capacity.

**Figure 2:**
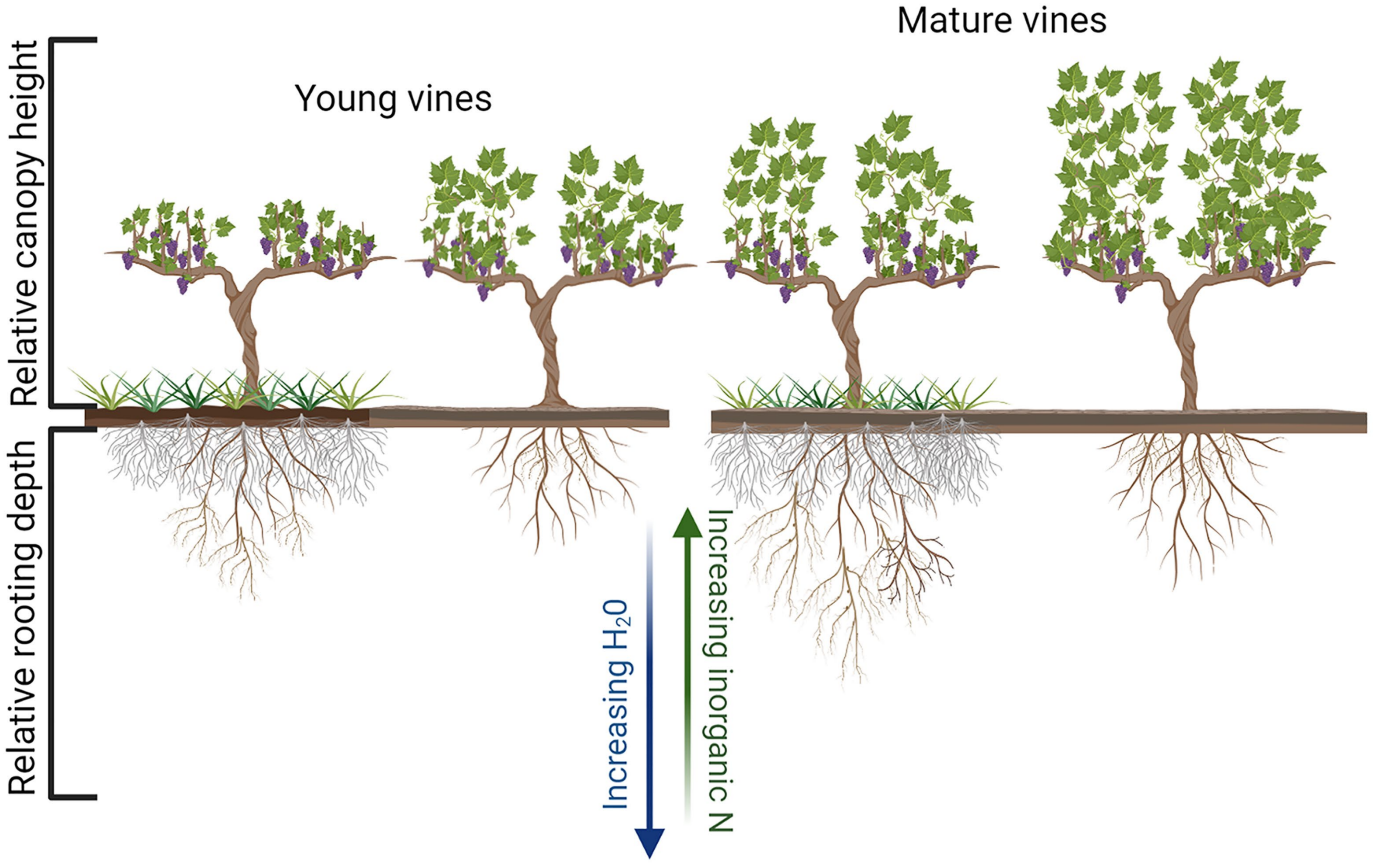
Diagrammatic representation of the impact of soil management, UVV vs. bare soil, on relative canopy height and rooting depth of both young and mature grapevines. Figure created with Biorender.com.

The influence of UVV on vegetative growth and yield varied across studies, vineyard sites, and years within the same site though some commonalities can be identified. For example, reductions in vegetative and reproductive growth induced by UVV are typically greater in younger vines than in older and more established vines (Figure 2), at least in regions with ample soil resources. Red fescue planted as an UVV in the fall of the second year of vineyard establishment induced yield and pruning weight reductions up to 39 and 46%, respectively, in the following growing season as compared to vines grown with herbicide-treated under-vine (Hatch et al., 2011). When white clover and natural vegetation were used as UVV in a young vineyard, pruning weight was reduced by approximately 50% by the fifth year, while yield was reduced by 16% in the natural vegetation treatment compared to plots maintained with under-vine herbicide (Karl et al., 2016b). Results from these studies and other work (Hatch et al., 2011; Fleishman et al., 2019) suggest that UVV can be used in the early years of a vineyard to favorably limit vine size at sites with high growth potential, although such significant vegetative growth and yield reductions may be considered an ecosystem disservice depending on the production goals of the grower. When UVV is implemented in older vineyards (10 years of age or more) the impact on vine size and yield has been considerably less (Giese et al., 2014; DeVetter et al., 2015; Jordan et al., 2016), likely due to more developed root systems which are able to access enough water and nutrients to maintain growth (Figure 2).

Another trend observed across studies is the more pronounced reduction in vegetative rather than reproductive growth when UVV is used (Giese et al., 2014; DeVetter et al., 2015; Hickey et al., 2016; Karl et al., 2016b; Chou and Vanden Heuvel, 2019), resulting in an increased Ravaz index (calculated as yield-to-pruning weight ratio). In one study, yield of vines growing with UVV was actually significantly higher than that of vines in the bare soil under-vine plots in a drier than average season (Chou and Vanden Heuvel, 2019). It is plausible that water content was higher in soil covered by UVV than in bare soil exposed to cultivations as reported in a previous work (DeVetter et al., 2015); if UVV is not actively growing it might form a green mulch and reduce loss of water *via* evapotranspiration.

### Grapevine-UVV Spatial Interaction

Belowground investigations can help better understand how grapevine and UVV interact to predict aboveground effects. Although studying root dynamics and functions present logistic and labor challenges, understanding how grapevine root systems respond and acclimate to UVV may help explain the variability, but also commonalities, of aboveground effects reported in the previous work.

The adoption of UVV creates a greater and more direct interaction between the root systems of the perennial (grapevines) and herbaceous (cover crop) species compared to using inter-row cover crops only, which could result in a greater resource uptake limitation and aboveground effects. When cover crops or natural vegetation grow in the inter-row only, the grapevine root system mainly colonizes the under-row area while the cover crops the inter-row, suggesting a compartmentalization of space and resources (Celette et al., 2005). In drier climates, this spatial separation might limit the access of grapevine roots to water and nutrients to a point that decreases vine vegetative growth (Celette et al., 2008, 2009). However, in climates with high precipitation and fertile soils, resources in the under-row area are often sufficient to support ample vine vegetative growth; therefore, the more direct spatial interaction imposed by UVV might be necessary to limit excessive grapevine vegetative growth. In a vineyard with complete cover crop floor coverage (inter- and under-row) soil volume available for grapevine root growth could be very limited; however, grapevines are considered plastic plants with one of the deepest rooting patterns (Smart et al., 2006) and in deep soils they could shift root distribution below the soil layers colonized by UVV.

A common belowground response to UVV, which was also reported in other fruit crop systems (Yao et al., 2009; Atucha et al., 2013), is a reduction of root production in shallow soil layers (20 cm), defined as a zone of high nutrient competition (Centinari et al., 2016; Klodd et al., 2016; Fleishman et al., 2019, 2021; Figure 2). Lower root growth in the top 20–30 cm of soil could reduce vine access to nutrients, as shallow soil strata have typically greater nutrient availability compared to deeper soil, which instead have higher soil water content (Klodd et al., 2016). This root distribution response can be described as inter-plant avoidance (Maina et al., 2002) and it was observed in both young and mature vines exposed to UVV from the year following vineyard planting (Klodd et al., 2016; Fleishman et al., 2019). The spatial segregation response reported in several studies is not surprising; some of the UVV species used, such as grasses, typically have a much denser root system compared to grapevines; and grass root length density (RLD; cm root/cm^3^ soil) can be up 10 times that of young grapevines (Fleishman et al., 2019). However, vines exposed to UVV later on, when in full production, might (Centinari et al., 2016) or might not (Giese et al., 2016) show an avoidance response.

A smaller and/or deeper root system (both as a proportion of the whole root system and as absolute RLD) could decrease vine access to specific soil resources (e.g., N vs. water) which are not homogeneously distributed across the soil profile (Figure 2). The entity of these growth reductions could vary greatly depending on the soil environment and volume explored by the root system; thus, it is not surprising that the aboveground growth reductions were observed in some studies but not in others, as previously discussed. A deeper root system might also be a useful trait in regions with ample precipitation that occasionally experience extended drought periods. Vineyards in regions with high precipitation events are often unirrigated and if vines can rely on deep water they might better withstand reduced water availability than those with a more shallow root system. Benefits of UVV under variable seasonal water availability are still speculative, but future studies could explore UVV potential to stabilize grapevine growth responses to variable soil moisture availability.

### Grapevine-UVV Temporal Interaction

The seasonal dynamics of root growth can affect the temporal interaction between the UVV and grapevines. Although belowground grapevine phenology is less predictable and not strictly coupled to aboveground phenological phases (Radville et al., 2016), vine root production typically exhibits a unimodal trend, with a major flush of root growth between bloom and veraison (Comas et al., 2010). A moderate water deficit is typically desired after bloom to reduce excessive vine vegetative growth without affecting C assimilation. If UVV roots grow before grapevines reach their seasonal peak of root production, they might limit grapevine root growth in the under-vine area. New roots are mainly absorptive, responsible for resource uptake; therefore, decreased root production might restrict vine water and nutrient uptake resulting in beneficial reduction of vine growth. The extent of these aboveground growth reductions, however, is less predictable than results obtained through deficit irrigation strategies in dry climates. Regardless, UVV still offers an opportunity to alleviate detrimental effects of excessive precipitation.

Cover crops with different growth cycles (e.g., perennial species vs. summer annual species) might introduce competition at different times of the vine growth cycle. Cool-season grasses exhibit a growth pattern that parallels the fast spring vegetative growth of vines and can be more effective in suppressing primary shoot growth compared to summer annual cover crop species which are planted later in the season. Competition for resources during early stages of berry development may, however, decrease berry size and thus yield potential. If summer annual UVV competes later in the season with the grapevines they could decrease the duration of lateral shoot growth (Centinari et al., 2016) or, in other instances, have no impact on vine size (Jordan et al., 2016). In addition to different competition timing, annual cover crops exhibit a shorter growth cycle and have smaller root systems relative to perennial cover crops, thus they tend to be less competitive for soil resources, at least in the first couple of years of establishment (Centinari et al., 2016; Jordan et al., 2016). Previous work indicated that perennial UVV species, mainly cool-season grasses, tend to be more competitive and effective in reducing vegetative growth than annual species (Giese et al., 2015; Hickey et al., 2016). However, repeated establishment of annual UVV over the years might still deplete shallow soil of water and nutrients and induce belowground vine response (Centinari et al., 2016).

### Grapevine-UVV Interaction Can Alter Root Traits

In some instances, but not always, shifts in root distribution induced by UVV were coupled with changes in other root traits, which suggest a plastic belowground vine response. For example, absorptive roots of vines growing with annual UVV species, such as annual ryegrass (*Lolium multiflorum*) and buckwheat (*Fagopyrum esculentum*), had longer median life span than those in the under-vine bare soil plots (Centinari et al., 2016). Shifts toward deeper root distribution induced by UVV could explain these differences, as roots produced in deeper soils tend to live longer than those growing in shallow soils (Anderson et al., 2003; Centinari et al., 2016). Results differ when grapevine roots growing in the UVV plots and in the zone of major interaction (0–40 cm) were sorted depending on their proximity to a UVV root. Grapevine roots growing without neighboring UVV roots lived much longer (over 300 days) compared to those growing nearby UVV roots (106 and 72 days in neighborhoods of annual ryegrass or buckwheat, respectively). This suggests that vines growing with UVV may maintain roots longer in soil patches with lower competition pressure, while shedding those in high competition areas (near UVV roots) to optimize resource uptake strategy (Centinari et al., 2016).

Other studies explored UVV-induced responses of absorptive root traits which are typically associated with increased efficiency of nutrient uptake, such as production of absorptive roots with smaller diameter, greater root length to mass ratio (specific root length, SRL, cm/g), and greater branching intensity (Klodd et al., 2016; Fleishman et al., 2019). Effects of UVV on these root traits, however, were not consistent between sites. For example, when young Noiret (*Vitis* hybrid sp.) vines grafted either on 101-14 Mgt (*V. riparia × V. rupestris*) or Riparia (*Riparia gloire*) were exposed to UVV (red fescue) for 1 year, they were able to compensate for reduction of absorptive RLD in the shallow soil (0–20 cm) with greater root length in deeper soil (21–40 cm), which was described as a zone of lower competition compared to the 0–20 cm depth increment (Fleishman et al., 2019). The same vines grown with UVV also had higher SRL and lower absorptive root diameter. However, despite these observed belowground changes, UVV vines still had lower macronutrients (particularly N) concentration and content in vegetative tissues and fruit compared to vines maintained with under-vine herbicide. Reduction in nutrient uptake was likely the main cause of the lower pruning weight induced by UVV reported in this study. In contrast, mature Cabernet Sauvignon (*Vitis vinifera*) vines grafted on the same rootstock (101-14 Mgt) of Fleishman et al. (2019) and with the same UVV species for 7 years exhibited only modest reductions in vegetative growth and no apparent changes in root morphological traits (e.g., root diameter, SRL, and branching intensity) compared to vines in plots with herbicide-treated under-vine (Klodd et al., 2016). More studies are needed to confirm if these contrasting results are related to the age of the vines and/or length (years) of UVV-vine interaction.

### Grapevine-UVV Competition for Water and Nutrients

Limited water and nutrient uptake affect many metabolic processes. Growth processes (i.e., shoot growth and early season berry growth) are the most sensitive and first affected by water deficits and nutrient deficiencies. Reduction in nutrients in vineyards with UVV could be direct, through lower soil availability, or indirect, *via* reduced water availability which can decrease nutrient movement toward the roots by mass flow or diffusion (Tinker and Nye, 2000) and N mineralization (Celette et al., 2009).

**Figure 3:**
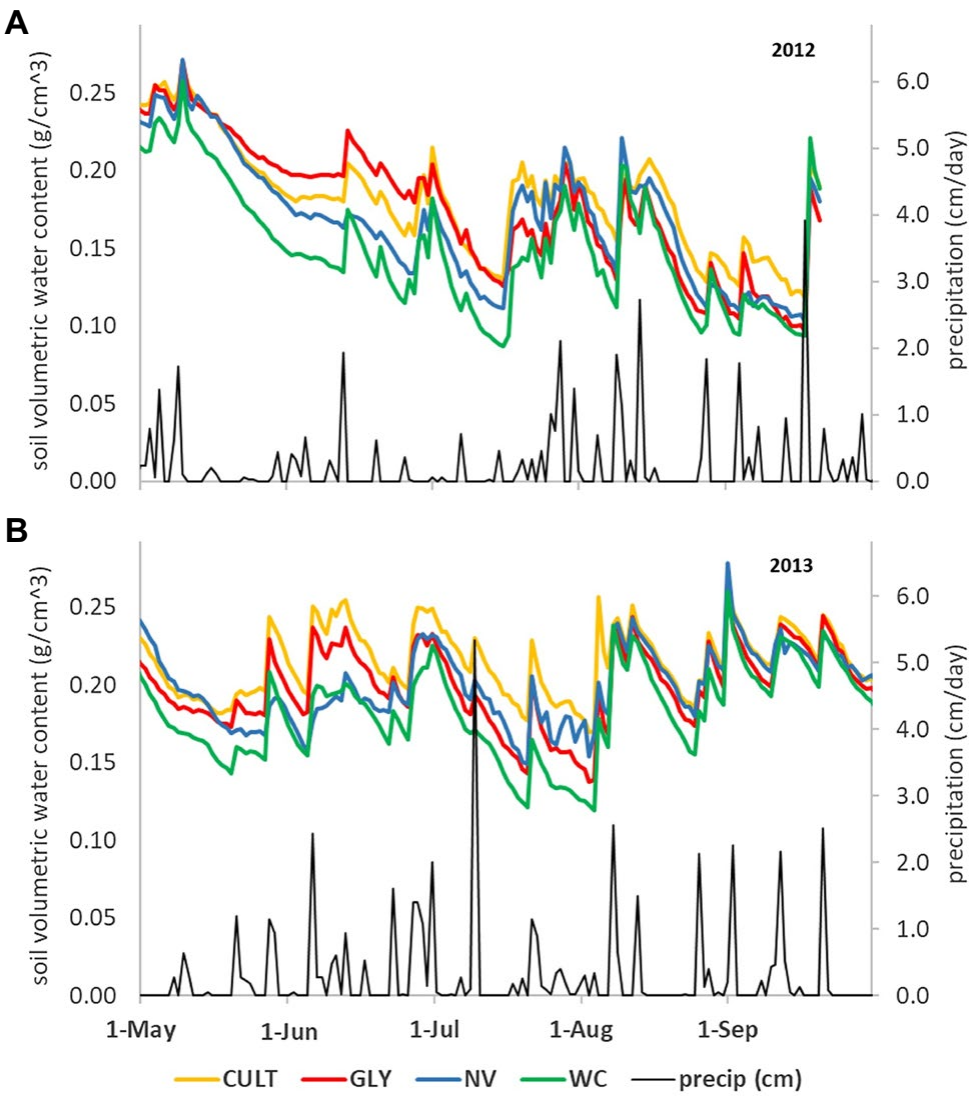
Soil water content (g/cm^3^) measured at midday under four UVV treatments in Lansing, NY, United States in **(A)** 2012 and **(B)** 2013 measured at 20 cm soil depth. CULT, cultivation; GLY, glyphosate; NV, natural vegetation; and WC, white clover. Data from Karl (2015), partially reported in Karl et al. (2016a).

Most work from regions with ample precipitation noted no or minimal competition for water in vineyards with UVV (Jordan et al., 2016; Klodd et al., 2016; Karl et al., 2016a; Fleishman et al., 2019). Only a few studies reported a positive correlation between decreased vine growth/yield and decreased soil moisture and vine water status (Hatch et al., 2011; Centinari et al., 2016). When examined across soil depths, UVV tended to decrease soil moisture below the zone colonized by perennial grasses (e.g., between 40 and 60 cm; Hickey et al., 2016; Klodd et al., 2016; Fleishman et al., 2019), but these differences were considered modest and did not affect the overall soil water storage. Soil moisture at shallow depths (0–20 cm) was reduced by one UVV species (white clover) planted annually in a vineyard in upstate NY but not by another UVV species (natural vegetation; Karl et al., 2016a). In both seasons of measurement, soil moisture differed among treatments until mid-summer as the vegetation established (Figure 3), but by veraison, there were no differences among treatments. These differences in shallow soil moisture (Figure 3) were not linked to differences in pruning weight (Karl et al., 2016b). Although UVV might affect soil moisture, there is no evidence that vines growing with UVV can adsorb water at deeper depths than those without UVV under wet weather conditions (Klodd et al., 2016; Fleishman et al., 2019). In general, it is hard to correlate soil moisture patterns with depth of water uptake in regions that do not experience prolonged dry-down periods because of the frequent and erratic rainfall events. The role of deep roots in water uptake might be more relevant in drier regions or seasons.

In regions where water availability is not typically a limiting factor, competition for nutrients might be more relevant. Compared to vines maintained with under-vine herbicide, UVV decreased plant N status (Giese et al., 2014; Fleishman et al., 2019) and extractable soil nitrates at shallow depths (<20 cm; Klodd et al., 2016) when perennial grasses were used. Competition for nutrients was less apparent when UVV species were planted on an annual basis (Karl et al., 2016b; Chou and Vanden Heuvel, 2019). However, interpretation of nutrient results among studies could be difficult due to different tissues (leaf petiole vs. blade) and methodologies used for collecting the tissue (e.g., time of the season and position on the canopy). Future studies should focus on a minimum of quantifying leaf blade nutrients to examine nutrient limitations since they are the most accurate tissue for measuring N and phosphorus (P) status of grapevines than leaf petioles (Schreiner et al., 2013).

It still unclear if decreased soil resource uptake induced by UVV competition has a meaningful impact the capability of a vine to assimilate C and on the allocation ratios among C sinks (e.g., shoot growth vs. fruit growth and ripening vs. storage in perennial tissues). Centinari et al. (2016) reported lower leaf C assimilation rate with the implementation of annual ryegrass under the vine in two of the 3 years of the study, but these observations were not always associated to lower leaf transpiration rate. Reduced C availability was listed as one of the potential causes of decreased grapevine fine root production for vines growing with annual ryegrass UVV. Hatch et al. (2011) reported lower C assimilation for vines in UVV plots compared to those managed with herbicide in only out of four dates, while leaf transpiration, stomatal conductance, and intercellular CO_2_ concentration of vines were unaffected by treatment. More research is needed in this area.

## Above and Belowground Acclimation Strategies

### Acclimation Strategies Could Depend on Vine Age

Interaction between a woody and herbaceous species is a dynamic process and acclimation strategies, although still understudied, might evolve over the years. Long-term studies (>5 years) of UVV in vineyards are needed to assess the sustainability of this practice across the lifetime of the vineyard. To date, we are aware of only two studies conducted in the humid eastern United States that assess aboveground vine response to UVV over 7 years (Giese et al., 2014; Hickey et al., 2016). In the first study (Giese et al., 2014), several cool-season perennial grasses were established in a vineyard already in full production (6 years after vineyard planting), while in the second trial (Hickey et al., 2016), a cool-season perennial grass was established under young vines (2 years after vineyard planting). At both sites, UVV favorably reduced vegetative growth compared to vines maintained with under-vine herbicide, but when UVV interacted with mature vines the reduction in pruning weight did not diminish or increase over time and appeared to be mainly driven by seasonal weather conditions. Additionally, there was no indication of root redistribution (Giese et al., 2016). In contrast, when vines were exposed to UVV starting at a young age, an acclimation to UVV competition was observed over the years. Differences in dormant pruning weight between vines in UVV and herbicide-treated plots diminished over 6 years and were mainly attributed to larger relative increases in size of UVV vines compared to vines maintained with under-vine herbicide over time (Hickey et al., 2016). Yield differences between under-vine management treatments also disappeared over time. It is plausible that in regions with ample precipitation and soil depth, young vines are able to acclimate to UVV competition over the years to a point that they can maintain or have limited reduction in aboveground growth despite having a much smaller absorptive root system than vines in bare soil under-vine plots (Klodd et al., 2016).

In addition to investigating shifts in root growth and morphological traits in response to UVV, as described earlier, several studies explored grapevine root association with beneficial microbes as a vine acclimation strategy to UVV. To date, investigations were mainly focused on arbuscular mycorrhizal fungi (AMF); however, other root-associated microbes that impact vine functioning might be affected by UVV as well. The guiding hypothesis was that vines growing with UVV would have greater AMF colonization than those growing with bare soil under-vine to improve efficiency of nutrient absorption especially in deeper soil layers not colonized by UVV roots. This could provide the vines with enough resources to maintain growth in competitive soil environments. However, to date, there is no indication that vines increase AMF colonization in response to UVV across the soil profile (0–100 cm) of young and mature vineyards (Klodd et al., 2016; Fleishman et al., 2019).

### Root System Genotype Might Affect Vine Response and Acclimation

Grapevine interaction with UVV over time can also be influenced by the root system genotype, but strong evidence is still lacking. Rootstocks are usually classified from low- to high-vigor based on their influence on the scion vegetative growth. If high-vigor rootstocks have greater RLD and higher soil water and nutrient depletion than low-vigor rootstocks they could more readily acclimate to belowground UVV competition. They might also be able to explore deep soil layers faster (both as a proportion of the whole root system and as absolute RLD) and therefore use more water in deeper soil too. These root traits could lead to a different aboveground response to UVV, such as less relative growth reduction for a grapevine grafted on a high-vigor compared to low-vigor rootstock. We could also speculate that, while differences between rootstocks might be exacerbated by competition with UVV, they would also diminish over time if the vines are able to acclimate to competition.

Two studies conducted at the same site examined below and aboveground responses to UVV competition of young grapevines (Noiret) grafted on two rootstocks that are considered to impart low (*Riparia*; *Riparia Gloire*) or moderate (101-14 Mgt) vigor, 1 year after UVV establishment and 2 years later (Fleishman et al., 2019, 2021). In general, the young low-vigor and medium-vigor rootstocks had a similar root redistribution response 1 year after UVV establishment. In response to UVV, both rootstocks had lower RLD in the shallow, high nutrient competition soil depth (0–20 cm) and greater RLD in the deeper, lower competition zone (21–40 cm). In contrast, 2 years later the medium-vigor rootstock displayed a more plastic belowground response to UVV competition than the low-vigor rootstock (Fleishman et al., 2021). While both rootstocks markedly and similarly decreased total root mass density (mg/cm^3^ of soil) between 0 and 20 cm, at deeper depths only the medium-vigor root system was influenced by the presence of UVV. These results suggest that root system genotypes might differ in their response to UVV competition, but that it might take a few years to observe significant differences. However, it is still unclear if differences in belowground rootstock-UVV interaction will lead to significant changes in aboveground growth.

## Practical Considerations for Farm Adoption

**Figure 4:**
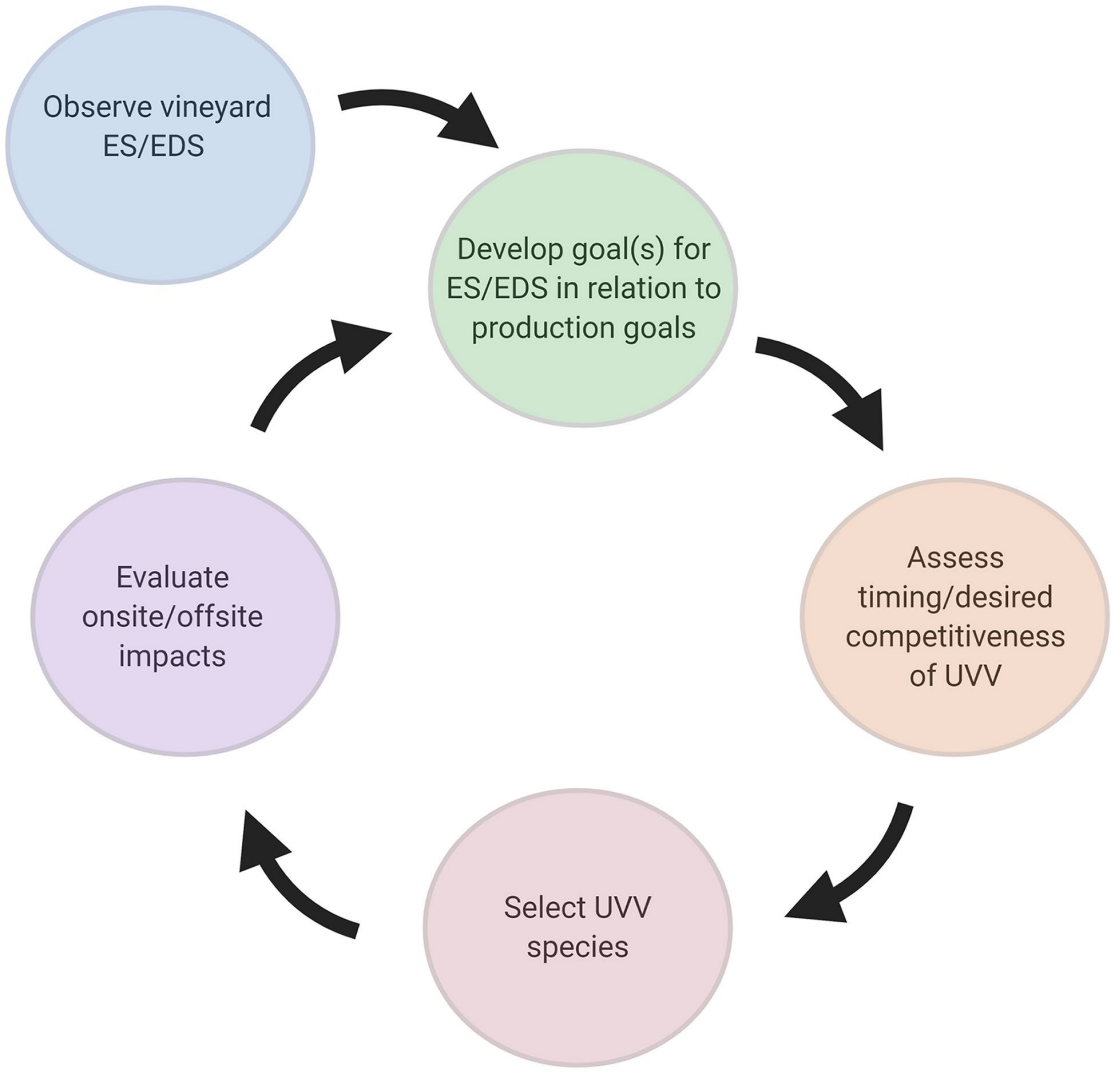
Iterative framework for adoption of UVV. ES, ecosystem services; EDS, ecosystem disservices. Figure created with BioRender.com.

Adoption of UVV in vineyards will require a flexible management plan due to both inter- and intra-annual variation in weather conditions (i.e., temperature and precipitation) and the practitioner’s production goals. The balance between ecosystem services and disservices provided by UVV is dynamic and there are a large number of factors that will influence vine response to UVV. Practitioners should carefully consider these factors if deciding to adopt UVV (Figure 4). While factors influencing vine response acclimation to UVV were examined above, they are discussed here in the specific context of their influence on adoption in commercial vineyards.

### Species of UVV

Studies on implementation of UVV in environments with high precipitation have investigated both perennial and annual species. In colder climates, annual species were selected since soil is generally hilled above the graft union for winter protection of scion buds on the trunk. Annual species are then replanted after the hill is pulled down in the spring. As a function of the hilling/unhilling, UVV in these vineyards will potentially compete with the vine for a shorter period of time compared to perennial UVV.

Choice of species will determine the competitiveness of the UVV within the context of the site and season. Species range not only in their competitiveness for water and nutrients, but also in the timing of when they are most competitive. For example, natural vegetation (i.e., managed weed growth) will generally provide almost season-long competition as a function of the diversity in species in the stand (Karl, 2015) although the species that comprise the stand will change by site and year (Jordan, 2014). In contrast, buckwheat establishes early in the growing season (prior to grapevine bloom in the northeastern United States) by easily out-competing weeds and then provides little competition for water and nutrients (Centinari et al., 2016) while chicory can provide intense competition that significantly reduces vine growth through the spring and summer (Jordan, 2014; Chou and Vanden Heuvel, 2019). Perennial grasses can provide enough competition to reduce growth rate throughout the season (Giese et al., 2015) without affecting wine water status in a region with high precipitation (Giese et al., 2014).

Legumes can release N depending on management, which can be an ecosystem service or disservice depending on production goals (Wise and Walter-Peterson, 2018). Release of N from a leguminous cover crop into nearby waterways (Karl et al., 2016a) is clearly an ecosystem disservice; however, the risk of N leaching from various legumes planted as UVV is unknown. Timing and amount of N release from a legume UVV were unpredictable both throughout the growing season and among seasons (Karl et al., 2016a). As the pruning weight of vines with white clover UVV was 30–57% lower than vines maintained with the under-vine herbicide, it is unlikely that a considerable amount N release from the white clover UVV was uptaken by the vines to support their growth (Karl et al., 2016b). The criteria for choosing species of UVV should primarily be based on timing and amount of desired competition with the vine, ability to establish and grow in the climate, desired height, and ease of management.

### UVV Planting and Management

Planting of UVV can be accomplished by hand or mechanically (Wise and Walter-Peterson, 2018; Wolf and Giese, 2020). Mowing is generally required unless dwarf species are well established and can be completed with a dedicated under-vine mower or a combination of row middle mowing and weed whacking (Wise and Walter-Peterson, 2018; Wolf and Giese, 2020). Tall or climbing weeds that reach the grapevine canopy can block sunlight from fruit, potentially reducing ripening and interfering with harvest. Location of the fruiting zone is impacted by the training system and will dictate the need to mow UVV and/or weeds.

As UVV can compete with vines, water and nutrient status must be carefully monitored through the season to ensure vines have the required resources (Wise and Walter-Peterson, 2018). The impact of UVV on pest pressure has not been studied, although Wolf and Giese (2020) warn of potential vole and cutworm damage to vines if vegetation is thick around grapevine trunks.

### Factors Affecting UVV Competitiveness and Vine Acclimation

Both vineyard and environmental factors will impact the competitiveness of the UVV with the vine as well as the ability of the vine to adapt to the competitive environment. These factors include vine vigor, vine age, soil properties, and soil nutrient and water availability.

Vigorous vines can withstand greater competition from UVV as vegetative growth of the vine tends to be reduced prior to reproductive growth (Chou and Vanden Heuvel, 2019; Fleishman et al., 2019). Presumably a function of rooting depth and volume as well as carbohydrate and nutrient storage capacity of permanent vine structures (cordons, trunk, and roots), the vegetative growth and reproductive growth of young vineyards are more impacted by UVV; as the vines mature the impacts of UVV are lessened (Hickey et al., 2016).

The physical, chemical, and biological characteristics of the soil will mediate competition for water and nutrients between the vine and the UVV. Soils lower in SOM will provide fewer nutrients and water holding capacity will be reduced compared to high SOM soils, potentially resulting in greater competition between the vine and UVV. Soil depth will impact the ability of the vine root systems to explore a greater soil volume in response to UVV competition, impacting vine nutrient and water status (Kallas, 2017). In vineyards with high SOM and deep soils, competition for resources by UVV is often an ecosystem service.

### Adjusting Management Practices

Both water and nutrient management plans may need to be adjusted with the adoption of UVV although interventions and timings of those interventions will be dependent on soil and environmental conditions. Pre-bloom irrigation may be necessary with the adoption of UVV in drier climates (Coniberti et al., 2018c). In climates with higher precipitation, nutrient additions may be needed, particularly during the critical phase of bloom through veraison (D’Attilio, 2014). Alternate forms of nutrient additions that are less dependent on water uptake – such as foliar applications – should be considered to offset nutrient deficiencies (D’Attilio, 2014). Use of a leguminous UVV cannot reliably increase N concentrations in vine vegetative tissue (Karl et al., 2016a) as sometimes only a small proportion of N from decomposing legumes is taken up by the vine (Brunetto et al., 2011). Wise and Walter-Petersen (2018) characterize the release of N from a clover used as a UVV as unpredictable.

### Impacts on Fruit Composition and Wine Sensory Perception

The impact of UVV on fruit composition and wine sensory perception would likely be indirect, with flavor and aroma compounds potentially impacted by plant adaptation to UVV through changes in berry size, leaf area to fruit ratio, fruiting zone microclimate (light exposure and temperature), and soil resource availability. The ability to study the impact of UVV on wine sensory characteristics has been hampered by the use of laboratory-style winemaking practices (i.e., chaptalization to standard sugar levels and lack of oak) as opposed to commercial fermentations. Nonetheless, a handful of studies have investigated the impact of UVV on consumers’ ability to differentiate resulting wines but the results are inconsistent among years and studies (Jordan et al., 2016; Karl et al., 2016b; Coniberti et al., 2018a).

### Cost of Adoption

Adopting and maintaining UVV includes the following potential costs: site preparation, seed, planting, mowing, and additional irrigation and fertilization. However, savings for producers may be realized through elimination of herbicide application and/or cultivation as well as reduced need for canopy management (e.g., hedging). Labor costs of establishment and maintenance of under-vine bare soil compared to UVV are difficult to gage as it depends on the cover crop species (i.e., cost of seeds and rate of seeding) used and its management needs, such as number of herbicide applications, cultivations, or mowing practices. Specific information on the cost of adoption and maintenance of UVV is sparse. Karl et al. (2016b) estimated that the cost of adoption and maintenance of UVV was around $84 and $169 per hectare for natural vegetation and white clover, respectively, compared to herbicide (glyphosate) and cultivation which was $548 and $1,036 per hectare, respectively.

Reduction in vegetative growth induced by UVV might require less labor-intensive canopy management practices, such as leaf removal and hedging. Labor savings can be hard to quantify, but a study conducted on Cabernet Sauvignon in a humid climate indicated that vines growing with a perennial fescue as UVV had smaller canopies, which were hedged in about half of the time compared to vines maintained with under-vine herbicide which were more vigorous (Hill, 2017). Similarly, time needed for leaf removal was reduced by 28% in UVV plots compared to herbicide plots. Use of UVV, however, can have a negative impact on economic returns if yield is significantly reduced. When differing under-vine management practices were implemented in the third year after planting on Cabernet Franc vines in the Finger Lakes region of New York State, yield was diminished from 11.5 t/ha in vines maintained with an under-vine herbicide to 8.4 t/ha in vines growing with white clover as UVV (Karl et al., 2016b).

**Figure 5:**
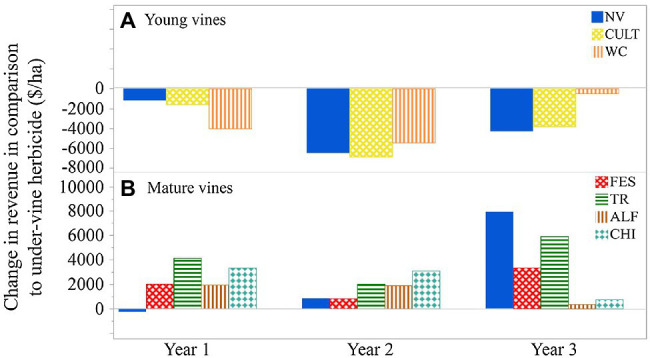
The impact of UVV on change in revenue in comparison with under-vine herbicide. **(A)** Young Cabernet Franc vines in New York State, data from Karl et al. (2016a). **(B)** Mature Cabernet Franc vines in New York State, data calculated from Chou and Vanden Heuvel (2019). NV, natural vegetation (weeds); CULT, cultivation; WC, white clover; FES, fescue; TR, tillage radish; AFL, alfalfa; and CHI, chicory.

A partial budget analysis could be used to estimate the financial implications of using UVV over more traditional under-vine management practices (Figure 5; Karl et al., 2016b). For example, when crop value per hectare was considered with the cost of planting and maintaining UVV, plots with under-vine herbicide had the highest economic return in a young vineyard (Figure 5A) when yield was reduced by early UVV implementation. However, when 15-year-old Cabernet Franc vines were subjected to three under-vine treatments over a three-year period in the same region, yield was either unaffected or increased significantly through the use of UVV, resulting in a positive impact on revenue per hectare (Figure 5B). While partial budgeting suggests that use of UVV has the potential to increase economic returns, additional benefits may arise from the ability for producers to market their wines with sustainability characteristics for quality differentiation (Schaufele and Hamm, 2017). Recent research suggests that consumers’ willingness to pay for wines may increase if a certification that takes into account vineyard biodiversity is on the bottle (Mazzocchi et al., 2019); wine consumers might also not be dissuaded by a modest price increase ($1 per bottle) if the wine was made with environmentally friendly farm practices, such as UVV (Kelley et al., 2017). Further analyses that consider economic costs and returns to the producer are needed to encourage adoption of UVV in appropriate regions.

## Conclusion

**Figure 6:**
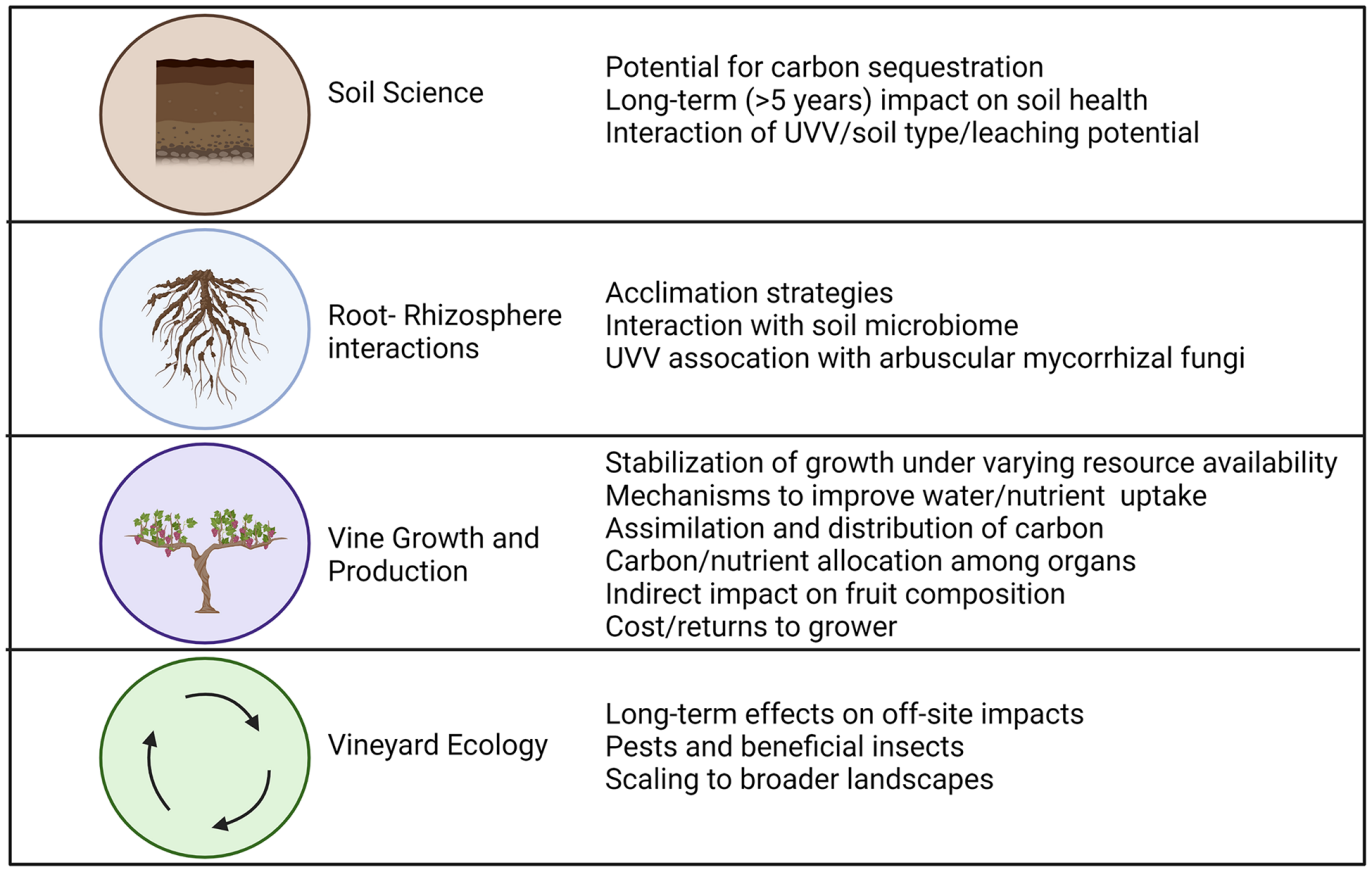
Knowledge gaps and research needs relating to the use of UVV in vineyards. Figure created with Biorender.com.

Adapting management strategies in the face of climate change are critical for maintaining and improving wine grape production worldwide. Several studies have demonstrated the potential of UVV to preserve soil health in grape growing regions with fertile soil and increasingly excessive precipitation, while also reducing herbicide input and excessive vine growth. This review discussed progress in several research areas which could help explain effects on vine growth and production and vineyard ecosystems (Figure 6). Many UVV species trialed to date can improve several parameters of soil health although long-term (>5 years) effects are still unknown. UVV effects on vine growth and productivity remain less predictable, but some similarities in vine responses to UVV competition have been identified across studies, such as a stronger reduction in vine vegetative rather than reproductive growth and greater UVV effects on vine nutrient rather than water status in regions with ample precipitation. There is also growing evidence of an age-dependent response of vines to UVV competition and that vines are able to redistribute their fine roots to areas of lower competition that are not highly colonized by UVV roots.

To promote widespread adoption of UVV in this changing climate, practitioners need guidelines on under-vine management options that best serve their production goals while maximizing the number of ecosystem services provided. Numerous knowledge gaps still exist which might prevent practitioners from more clearly predicting vine response and acclimation to UVV over the years. Figure 6 summarizes important research needs that were identified throughout the review. Although they were classified by discipline or research area, the research needs are cross-disciplinary with required approaches spanning from soil science through plant ecophysiology to crop production to address these knowledge gaps. A transdisciplinary approach is critical for linking shifts of root distribution in response to UVV to changes in soil environment (e.g., resources and microbiome), vine functioning, and fruit composition. For instance, an integrated approach will help clarify if deeper root systems of vines growing with UVV, which has been reported in several studies, can stabilize vine productivity under more erratic rainfall patterns (i.e., more intensive rainfall alternated by dry periods) associated with climate change. Furthermore, there is little evidence that vines growing with UVV can improve efficiency of resource uptake by modifying morphological root traits. Work in this area is still limited with contrasting results likely due to differing vine age, root system genotype (i.e., rootstock), and the time of vine UVV coexistence, among other reasons. Future work should explore if grapevines exhibit mechanisms which will improve nutrient or water uptake capacity in a highly competitive soil environment, and if these mechanisms evolve over the years, allowing vines to acclimate to the presence of UVV and maintain above ground growth and/or production despite a smaller root system.

## Author Contributions

JV and MC developed the concept and wrote the manuscript. All authors contributed to the article and approved the submitted version.

## Conflict of Interest

The authors declare that the research was conducted in the absence of any commercial or financial relationships that could be construed as a potential conflict of interest.

## Publisher’s Note

All claims expressed in this article are solely those of the authors and do not necessarily represent those of their affiliated organizations, or those of the publisher, the editors and the reviewers. Any product that may be evaluated in this article, or claim that may be made by its manufacturer, is not guaranteed or endorsed by the publisher.
